# Plant functional diversity enhances associations of soil fungal diversity with vegetation and soil in the restoration of semiarid sandy grassland

**DOI:** 10.1002/ece3.1875

**Published:** 2015-12-29

**Authors:** Xiaoan Zuo, Shaokun Wang, Peng Lv, Xin Zhou, Xueyong Zhao, Tonghui Zhang, Jing Zhang

**Affiliations:** ^1^ Naiman Desertification Research StationCold and Arid Regions Environmental and Engineering Research InstituteChinese Academy of SciencesLanzhou730000China; ^2^ Laboratory of Stress Ecophysiology and Biotechnology (LSEB)CAREERICASLanzhou730000China

**Keywords:** Ecosystem properties, functional dispersion, functional traits, mass ratio hypothesis, niche complementarity, structure equation model, vegetation restoration

## Abstract

The trait‐based approach shows that plant functional diversity strongly affects ecosystem properties. However, few empirical studies show the relationship between soil fungal diversity and plant functional diversity in natural ecosystems. We investigated soil fungal diversity along a restoration gradient of sandy grassland (mobile dune, semifixed dune, fixed dune, and grassland) in Horqin Sand Land, northern China, using the denaturing gradient gel electrophoresis of 18S rRNA and gene sequencing. We also examined associations of soil fungal diversity with plant functional diversity reflected by the dominant species' traits in community (community‐weighted mean, CWM) and the dispersion of functional trait values (FD_is_). We further used the structure equation model (SEM) to evaluate how plant richness, biomass, functional diversity, and soil properties affect soil fungal diversity in sandy grassland restoration. Soil fungal richness in mobile dune and semifixed dune was markedly lower than those of fixed dune and grassland (*P *<* *0.05). Soil fungal richness was positively associated with plant richness, biomass, CWM plant height, and soil gradient aggregated from the principal component analysis, but SEM results showed that plant richness and CWM plant height determined by soil properties were the main factors exerting direct effects. Soil gradient increased fungal richness through indirect effect on vegetation rather than direct effect. The negative indirect effect of FDis on soil fungal richness was through its effect on plant biomass. Our final SEM model based on plant functional diversity explained nearly 70% variances of soil fungal richness. Strong association of soil fungal richness with the dominant species in the community supported the mass ratio hypothesis. Our results clearly highlight the role of plant functional diversity in enhancing associations of soil fungal diversity with community structure and soil properties in sandy grassland ecosystems.

## Introduction

Soil fungi play the crucial role in determining nutrient cycling in terrestrial ecosystems (Deacon et al. 2006; Bardgett and van der Putten 2014). Soil fungal diversity and its determinants are of utmost importance to understand ecosystem functioning (Lentendu et al. 2011). Changes of soil fungal richness at the global scale are strongly affected by the combination of climatic factors, latitude, and soil properties (Tedersoo et al. 2014). Vegetation compositions and soil properties are thought to exert important effects on soil fungal composition and diversity at local and regional scales (Burke et al. 2009; Siciliano et al. 2014; LeBlanc et al. 2015; Sterkenburg et al. 2015). The trait‐based approach can clearly reveal effects of plant functional diversity on ecosystem function and properties (Petchey and Gaston 2006; Lienin and Kleyer 2012; Lavorel 2013). However, the empirical studies on relationships between soil fungal diversity and plant functional diversity are still limited (Lavorel 2013; Moreau et al. 2015).

Different plants differ in provision of photosynthetic carbon for soil fungal growth, which allows plant communities to shape soil fungal communities, thus affecting soil fungal diversity (Wardle 2006; Dickie 2007). Increasing plant richness increases soil fungal richness in grassland (LeBlanc et al. 2015) and rainforest ecosystems (Peay et al. 2013), because the diversity of litter increases the diversity of carbon compounds and other resources for soil fungal communities. Studies of the effects of plant biomass on soil fungal richness have shown inconsistent findings, including positive (Wagg et al. 2011) and negative effects (Hiiesalu et al. 2014). Concurrently, many studies have also suggested that soil fungal diversity is strongly dependent on soil properties (Prevost‐Boure et al. 2011; Siciliano et al. 2014; LeBlanc et al. 2015). Soil fungal richness increased with increasing soil fertility (i.e., organic matter and nitrogen) (Lauber et al. 2008; Siciliano et al. 2014; Sterkenburg et al. 2015) and soil water content (Drenovsky et al. 2004). Thus, untangling the response of soil fungal diversity to changes of vegetation and soil properties will improve our understanding of how biotic and abiotic factors affect the soil fungal community, which will be helpful for management of natural ecosystems.

Numerous studies have shown that ecosystem processes related to carbon (C) and nitrogen (N) cycling are driven by plant functional diversity reflected by traits of the most abundant species (community‐weighted mean, CWM) and the variety of functional trait values (Butterfield and Suding 2013; Lavorel 2013). CWM expressing the dominant trait value of most abundant species was defined as the abundance‐weighted mean trait value for a community (Violle et al. 2007; Butterfield and Suding 2013). CWM traits were found to explain species richness (Le Bagousse‐Pinguet et al. 2014) and soil microbial community composition in natural grasslands (de Vries et al. 2012). The CWM effects may primarily be attributed to the biomass ratio hypothesis (Laughlin 2011; Conti and Díaz 2013; Lavorel 2013). Yet, the high functional dispersion (FDis) may reflect an increase in complementarity in resource use among species in plant community, thus enhancing ecosystem function (Schumacher and Roscher 2009), which supports the niche complementarity hypothesis (Tilman 1997; Lavorel 2013). In addition, some studies have clearly revealed that soil properties strongly affect plant functional traits and diversity (Bernard‐Verdier et al. 2012; Jager et al. 2015). However, information concerning direct or indirect associations of soil fungal diversity with plant functional diversity controlled by soil properties in natural ecosystems is still lacking.

The Horqin sandy grassland is located in the semiarid area of southeastern Inner Mongolia, northern China. This region has suffered severe desertification since the early 1950s, due to long‐term overgrazing and extensive crop cultivation (Zhao et al. 2005, 2006). The original landscape dominated by grassland with scattered trees (mainly elms, *Ulmus* spp.) has also been mostly replaced by sandy dunes (Zhang et al. 2005; Zhao et al. 2005). Thanks to the annual precipitation of 350–500 mm, most mobile dunes can gradually be stabilized to semifixed or fixed dunes after excluding grazing for approximately 15 or 30 years, respectively (Zhang et al. 2005; Liu et al. 2009b; Li et al. 2012). Vegetation succession occurs from the sand pioneer in mobile dune to the low shrub communities in semifixed dune then toward the annual herb‐dominated communities in fixed dune (Zhang et al. 2005; Zuo et al. 2009). Species richness, biomass, soil C, total N, very fine sand, silt, and clay increase following sandy grassland restoration (Qiao et al. 2012; Zuo et al. 2012b). Soil properties strongly affect plant compositions and distributions along the restoration gradient of sandy grassland (Zuo et al. 2009, 2012a). Previous studies have shown that sandy vegetation restoration may affect soil microbe composition and diversity (Wang et al. 2011; Jiang et al. 2014); however, little is known about how plant community structure and soil properties affect soil fungal diversity in sandy grassland restoration.

Although soil microbial communities have effects on plant diversity and productivity (van der Heijden et al. 2008), most studies have focused on effects of plant species or compositions on soil microbial communities (Burke et al. 2009; Peay et al. 2013; LeBlanc et al. 2015). Due to the complex plant–microbe interactions, our study only considers effects of vegetation structure and soil properties on soil fungal diversity in sandy grassland restoration. In particular, we applied the structural equation model (SEM) to examine whether plant functional diversity can enhance associations of soil fungal diversity with vegetation structure and soil properties. We tested the three hypotheses: (1) soil fungal diversity was determined by soil gradient; (2) soil fungal diversity was associated with plant richness; and (3) plant functional diversity direct or indirectly affected soil fungal diversity in sandy grassland restoration.

## Methods

### Site description

This study was conducted in southwest of Horqin Sandy Land (42°55′N, 120°42′E; 360 m elevation), Inner Mongolia, Northern China. The climate is continental semiarid with a warm summer and a very cold winter. The annual mean temperature is around 6.4°C, and annual average precipitation is 360 mm. The soil is sandy chestnut soils with loose structure and vulnerable to wind erosion.

Within the study area of 32 km^2^, we selected 24 sites 0.5–8 km apart corresponding to the four typical habitat types in sandy grassland restoration, including mobile dune with <10% vegetation cover (MD), semifixed dune with 10–60% vegetation cover (SFD), fixed dune with more than 60% vegetation cover (FD) and grassland type with more than 60% vegetation cover (G) (Liu et al. 2009a; Zuo et al. 2012b). Each habitat type had six replicate sites. The sand pioneer plant, an annual forb of *Agriophyllum squarrosum* (*Linn*.) *Moq.,* is a dominant plant in mobile dunes. Semifixed dunes are dominated by *Artemisia halodendron Turcz. ex Bess* shrub and annual forb *of Corispermum macrocarpum Bge*. Fixed dunes are dominated by annual forb of *Artemisia scoparia Waldst. et Kit*. Grasslands are dominated by annual forb of *A. scoparia* and perennial grass of *Phragmites communis Trrin. Fund*. and *Pennisetum centrasiaticum Tzvel*. Semifixed dunes and fixed dunes were naturally restored from mobile dunes caused by long‐term overgrazing by excluding livestock since 1995 and 1980, respectively. Before the grazing exclusion, the landscape of these dune sites was characterized by areas with mobile dunes caused by long‐term overgrazing. Grassland sites were also restored using livestock exclusion (since 1996), but were naturally colonized by perennial grasses rather than shrubs or forbs.

### Sampling and measurement

Collection and measurement of sampling was carried out in mid‐August 2013. At each site, we established a homogeneous 20 × 20 m plot. Five 1 × 1 m quadrats were set up at the four corners and the center in each plot to carry out vegetation survey and soil sampling. Within each quadrat, the number of plant species and plant cover was recorded, aboveground plant biomass of each species was harvested, litter was collected, and then, the belowground roots were taken by three soil cores from the top 10 cm using an 8‐cm‐diameter auger. Three random soil samples at depth of 0–10 cm within the quadrat were collected using a 3‐cm‐diameter soil auger and pooled to form one composite sample for laboratory analysis. Concurrently, with the same auger, one soil core was taken to measure soil water content within the quadrat. Soil samples were also collected for soil bulk density using a soil auger equipped with a stainless‐steel cylinder (5 cm in both diameter and height).

Roots were washed and handpicked over a 1‐mm screen to remove all soil, pebbles, and debris. The aboveground biomass, litter, and roots were dried at 60°C for 48 h in laboratory. Soil particle size from international and USDA classification systems was determined by the wet sieving method (ISO, 1998). Soil pH and electrical conductivity (EC) were measured in a 1:1 soil–water slurry and in a 1:5 soil–water aqueous extract (Multiline F/SET‐3, WTW, Weilheim, Germany), respectively. Soil total C and N were analyzed by an elemental analyzer (vario Macro cube; Elementar, Hanau, Germany). Vegetation characteristics and soil properties of different habitat types are shown in Table S1.

### Plant functional traits and diversity

Functional traits associated with leaf and plant growth of the most abundant species (total of 34 species) were measured for 5–10 individuals of each species in each quadrat, including specific leaf area (SLA), leaf dry matter content (LDMC), leaf carbon and nitrogen ratio (C:N), and plant height (H). Plant traits of all samples were measured using the standard methodologies (Cornelissen et al. 2003; Conti and Díaz 2013; Kichenin et al. 2013). We also used single‐trait (CWM) and multitrait indices (function dispersion, FDis) to calculate functional diversity (Butterfield and Suding 2013; Spasojevic et al. 2014). CWM was simply calculated as the abundance‐weighted mean trait value for a community (Violle et al. 2007; Butterfield and Suding 2013): CWM (trait_*X*_) = Σ*p*_*i*_ *x*_*i*_, where CWM (trait_*X*_) is the CWM for a *X* trait, *p*_*i*_ is the relative biomass of the *i*‐th species in the community and *x*_*i*_ is the trait value of *i*‐th species. FDis was measured as the multiple traits dispersion within the functional volume of plant community (Laliberté and Legendre 2010) and regarded as a surrogate measure of functional richness and functional divergence (Schleicher et al. 2011). We also standardized the four traits to calculate FDis in order to avoid scale and unit effects (Casanoves et al. 2011). A summary of functional traits and diversity of four habitat types included in the study is provided in Table S2.

### Soil fungal diversity

Soil fungal diversity were conducted by means of the polymerase chain reaction with denaturing gradient gel electrophoresis (PCR‐DGGE) (Hoshino and Morimoto 2008; Fracetto et al. 2013; Si et al. 2013). One fresh composite sample from the top 10 cm was also collected from five quadrats in each plot to extract total soil DNA in laboratory. 18S rRNA genes were amplified with the fungal‐specific primers NS1 (5′‐GTA GTC ATA TGC TTG TCT C‐3′), GCfung (5′‐CGC CCG CCG CGC CCC GCG CCC GGC CCG CCC CCG CCC CAT TCC CCG TTA CCC GTT G‐3′), and fung (5′‐ CAT TCC CCG TTA CCC GTT G‐3′) (Hoshino 2012; Si et al. 2013). Amplification was performed in a solution containing approximately 50 ng of DNA, 5 *μ*L buffer of PCR 10x, 3.2 *μ*L of dNTP (2.5 mmol/L), 0.4 *μ*L of rTaq(5 U/*μ*L), and 1 *μ*L of each primer, and sterile ultrapure water was used to adjust the mixture to a final volume of 50 *μ*L. PCR amplification was run on a T‐gradient diversity system (Bio‐Rad, Segrate, Italy). The amplification was performed under the following conditions: an initial denaturation for 5 min at 94°C, amplification denaturation (94°C for 1 min), annealing (50°C for 45 sec), 30 cycles, extension (72°C for 1 min), and a final extension of 10 min at 72°C.

Polymerase chain reaction samples (10 *μ*L) containing amplicons were loaded onto the 1 mm thick 8% (w/v) acrylamide gel in 1 × TAE buffer using a denaturing gradient ranging from 35% to 55% (100% denaturing solution included 7 mol/L urea and 40% deionized formamide). Electrophoreses were performed at 60°C and 150 V for 4 h. The gel was stained in silver staining solution for 20 min and photographed to get DGGE profile band using a Bio‐Rad transilluminator (BIO‐RAD Laboratories) under UV light. Then, DGGE profile bands were analyzed by Quantity One software Bio‐Rad Laboratories, Inc.: Hercules, CA, USA (version 4.6.2). The fungal composition was presented using the DGGE profile bands and band intensity of Heatmap Illustrator (http://hemi.biocuckoo.org). Common bands (number of population) were carefully excised from the DGGE gel and subjected to sequencing, and then, the obtained sequences of the selected DGGE bands were identified using BLAST database in NCBI. Totally 38 bands were detected from four habitats of sandy grassland (Fig. S1). BLAST search showed that most of the sequences were closely related to fungal clones or isolates in GenBank (Table S3). Then, a phylogenetic tree using the neighbor‐joining method by MEGA 5 (Tamura et al. 2011) was built to identify closely related species and phylogenetic affiliations among the obtained sequences (Fig. S2). Species richness and evenness were calculated based on the bands number and band intensity in the DGGE (Mouillot et al. 2011; Fracetto et al. 2013).

### Data analysis

We firstly used PCA method to aggregate 11 soil properties from 24 plots and summarized the composite independent variables from PCA along the habitat gradient of sandy grassland restoration (Zuo et al. 2012a; Le Bagousse‐Pinguet et al. 2014). Intraset correlations from the PCA are used to assess the importance of environmental soil properties (Table S4). The first PCA axis, explaining 86.8% of the total variability, represented the main soil gradient which was significantly and positively correlated with soil C, N, C/N, pH, EC, very fine sand, slit and clay, and soil water content and negatively with soil bulk density and coarse sand. The first PCA axis was used in further analyses, specifically to test relationships among soil properties, community structure and soil fungal diversity. Here, we also used the correlation analysis to examine their relationships among soil properties, vegetation characteristics, and function diversity and then used the linear regression analyses to obtain the most important predictors of soil fungal diversity. Finally, we applied the SEM for determining the direct and indirect effects of the combination of factors on soil fungal diversity. SEM is an advanced multivariate statistical method to assess relationships among networks of variables which are able to act simultaneously as both predictor and response (Jonsson and Wardle 2010; Zuo et al. 2012a; Spasojevic et al. 2014; White et al. 2014). Starting from the most complex initial model that included all significant variables from plausible interaction pathways, model simplification was based on the significance of the regression weights (Jonsson and Wardle 2010; Mouillot et al. 2011). We removed the nonsignificant variables or paths to find the best model with the lowest Akaike information criterion (AIC) and assessed model fit with a chi‐squared tests, root‐mean‐square error of approximation (RMSEA), and goodness‐of‐fit index (GFI) (Lamb 2008; White et al. 2014).

All data were expressed as mean ± 1 SE (*n* = 6), and the analysis of variance (ANOVA) was across the plot level. All statistical analyses were performed by SPSS SPSS, Inc., Chicago, IL, USA (version 16.0). All functional diversity indices were calculated using the statistical package FDiversity v. 2011 (Casanoves et al. 2011). The structural equation modeling was applied using AMOS 20.0 software (Arbuckle 2009).

## Results

**Figure 1:**
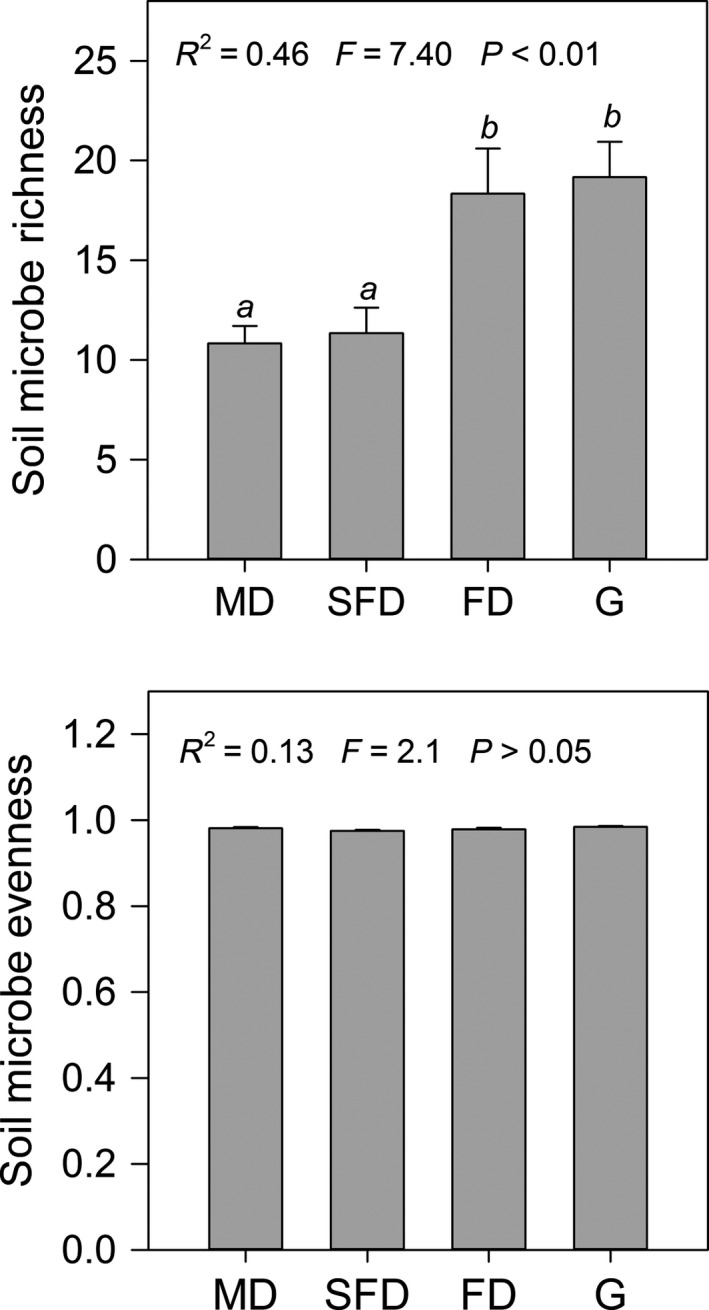
Comparisons of soil fungal richness and evenness at four habitats of sandy grassland (*n* = 6). Different letters in from mean values indicate statistical difference among different habitats at *P *<* *0.05.

Habitat changes had a significant effect on soil fungal richness (*P *<* *0.01), but no effect on fungal evenness (Fig. 1, *P *>* *0.05). Soil fungal richness in mobile dune and semifixed dune was significantly lower than those of fixed dune and grassland (*P *<* *0.05) and did not differ between mobile dune and semifixed dune and between fixed dune and grassland (*P *>* *0.05).

**Figure 2:**
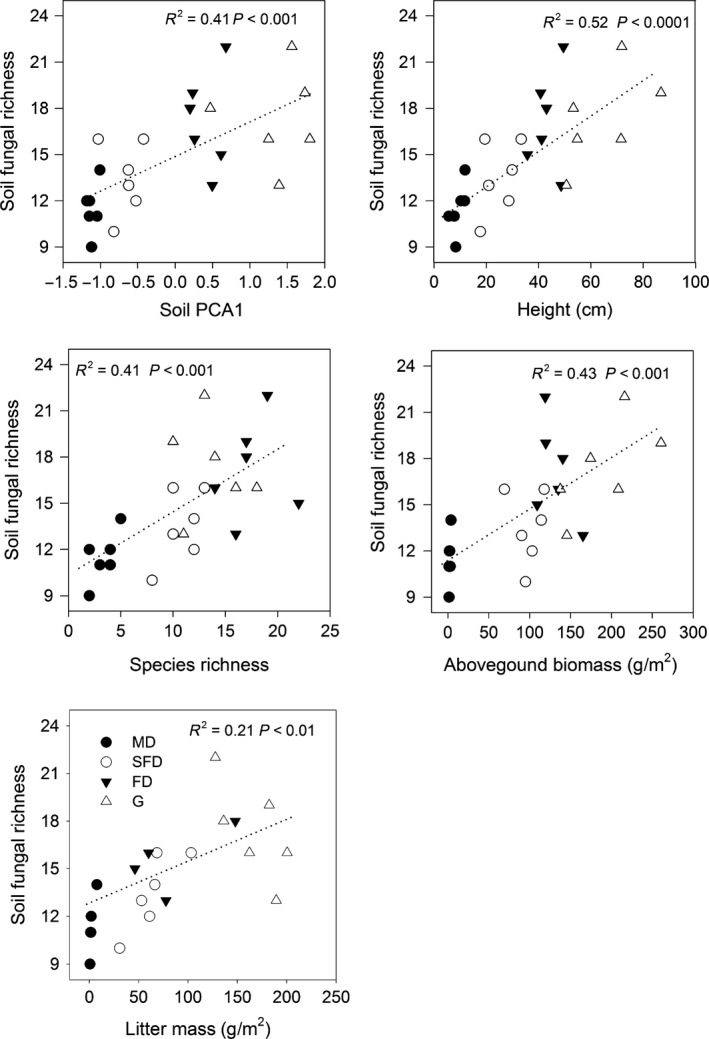
Simple linear regression analyses of soil PCA1, plant height, species richness, biomass, and litter mass with soil fungal richness in sandy grassland restoration (*n* = 24). MD, mobile dune; SFD, semifixed dune; FD, fixed dune; G, grassland.

Correlation analyses indicated that soil gradient aggregated from PCA had the significant and positive correlations with plant species richness, aboveground plant biomass, root biomass, litter mass, and functional diversity components (Table 1, P* *<* *0.05). Species richness, aboveground plant biomass, root biomass, and litter mass were significantly and positively associated with plant height, leaf C:N, and FDis (*P *<* *0.05). FDis was significantly and positively correlated with plant height and leaf C:N (*P *<* *0.05). We also found the significant and positive correlations among species richness, aboveground plant biomass and litter mass (*P *<* *0.05). Soil fungal richness was significantly and positively correlated to soil gradient, CWM plant height, species richness, plant biomass, and litter mass (Fig. 2, *P *<* *0.01).

**Table 1:** Correlation coefficients among soil PCA1, community‐level traits, vegetation characteristics, and function diversity in sandy grassland restoration (*n *=* *24)

	Soil PCA1	Height	SLA	LDMC	Leaf C:N	Species richness	Aboveground plant biomass	Root biomass	Litter mass	Litter C/N
Community‐weighted means (CWM)										
Height	0.93**									
SLA	0.52**	0.40								
LDMC	0.50*	0.36	0.37							
Leaf C:N	0.51*	0.57**	−0.01	−0.03						
Vegetation characteristics										
Species richness	0.68**	0.65**	0.37	0.15	0.65**					
Aboveground plant biomass	0.85**	0.96**	0.25	0.21	0.67**	0.69**				
Root biomass	0.43*	0.41*	−0.22	−0.03	0.67*	0.31	0.49*			
Litter mass	0.84**	0.85**	0.28	0.20	0.53**	0.54**	0.83**	0.65**		
Litter C:N	−0.40	−0.30	−0.38	−0.66**	0.14	−0.13	−0.15	0.03	−0.25	
Multitrait functional divergence index										
Function dispersion	0.53*	0.44*	0.06	−0.04	0.72**	0.42*	0.48*	0.59**	0.62**	0.10

SLA, specific leaf area; LDMC, leaf dry matter content, C:N, carbon‐to‐nitrogen ratio.

*P <* *0.05; **P <* *0.01; ***P <* *0.001.

**Figure 3:**
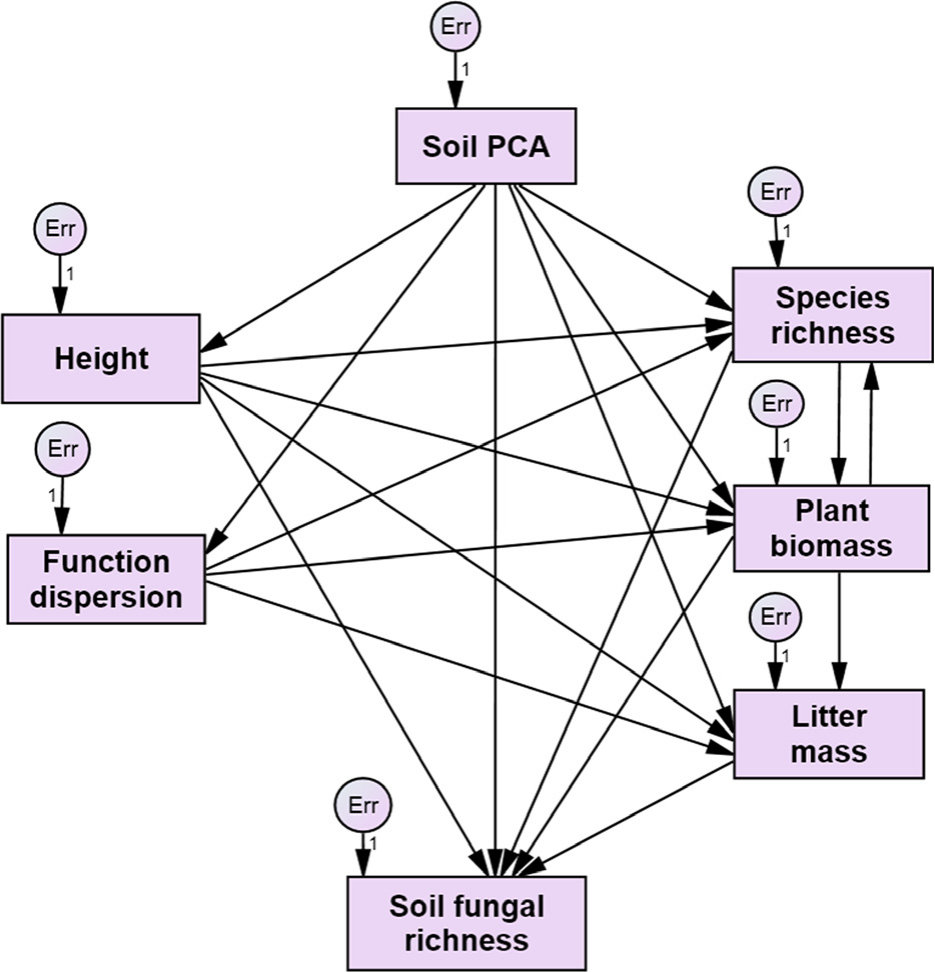
Initial structural equation model showing all interaction pathways of soil gradient, function diversity, species richness, plant biomass, litter mass, and soil fungal richness. Single‐headed arrows indicate paths. Double‐headed arrows show the correlations included in the model based on modifications proposed by AMOS (procedure modification indices). The exogenous unobserved variables err account for the unexplained error. Their regression weights were a priori set to unity.

**Figure 4:**
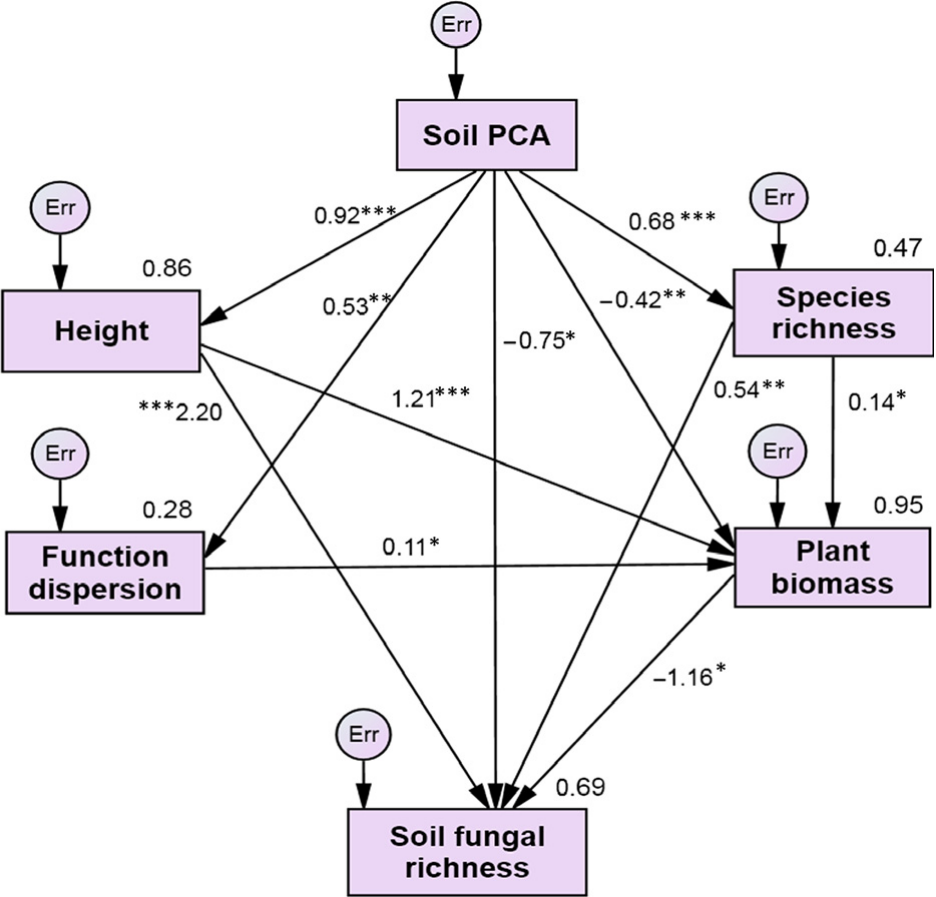
Final structural equation model with standardized direct effects. Standardized regression weights (along path) and total variance explained as a result of all predictors pointing to that variable (top right corner of rectangle). Single‐headed arrows represent direct effects. *, **, and *** indicate statistically significant paths at *P *<* *0.05, *P *<* *0.01, and *P *<* *0.001, respectively.

We created the initial SEM to show all interaction pathways of soil properties, species richness, plant biomass, litter mass, CWM plant height, FDis, and soil fungal richness (Fig. 3). The model including the CWM plant height, FDis, species richness, plant biomass, and soil gradient (*χ* ^2^ = 2.76, *P *=* *0.60; RMSEA = 0.00; GFI = 0.96) was the best fit to explain the variance in soil fungal richness (*R* ^2^ = 0.69, Fig. 4). Further, the model showed that soil gradient had the significant and positive effects on plant height, FDis and species richness (*P *<* *0.01) (Fig. 4, Table 2). Plant height and species richness had the positively direct effects on soil fungal richness (*P *<* *0.01) and plant biomass (*P *<* *0.05). The indirect effect of FDis on soil fungal richness was through the negative effect of plant biomass (Table 2). Soil gradient increased soil fungal richness through indirect rather than direct effects (Table 2).

**Table 2:** Direct, indirect and total effects on soil fungal richness based on standardized values of statistically significant SEM paths (*P *<* *0.05)

Predictor	Pathway to species fungal richness	Effect
Soil PCA	Direct	−0.75
	Indirect	1.41
	Total	0.66
Height	Direct	2.20
	Indirect	−1.40
	Total	0.80
Function dispersion	Direct	NS
	Indirect	−0.13
	Total	−0.13
Species richness	Direct	0.54
	Indirect	−0.16
	Total	0.37
Plant biomass	Direct	−1.16
	Indirect	NS
	Total	−1.16

NS, nonsignificant relationships.

## Discussion

Our study represents a first attempt to evaluate the role of plant functional diversity in effects of vegetation and soil properties on soil fungal diversity in sandy grassland restoration. Our results supported these hypotheses that soil fungal diversity was affected by soil gradient, species richness and plant functional diversity in sandy grassland restoration. Increasing species richness and CWM plant height controlled by soil gradient increase soil fungal richness. This is consistent with the finding that soil fungal richness is strongly dependent on plant community and soil properties in natural ecosystems (LeBlanc et al. 2015; Sterkenburg et al. 2015). Effects of plant richness and CWM plant height on soil fungal richness were direct rather than indirect, while soil properties, function dispersion, and plant biomass acted indirectly. Our results have suggested that the dominant species' trait reflected by CWM is one of important drivers of soil fungal diversity; however, niche complementarity as reflected by FDis is only importance for plant biomass. Most of results from the simple linear regression analyses were in agreement with SEM, but resulted in the oversimplification of conclusions. In comparison, SEM analysis provided the new insights on the underlying ecological relationships (Lamb 2008; White et al. 2014).

Not surprisingly, we found that plant richness positively affected soil fungal richness in sandy grassland restoration, supporting the other studies in which plant richness is a good predictor of fungal richness (Peay et al. 2013; LeBlanc et al. 2015). This is clearly explained by that changes of plant diversity will modify the resource availability and microclimate for the heterotrophic microbial communities in soil and thus will modify soil fungal composition (Zak et al. 2003; McGuire et al. 2012). However, the inconsistent relationships between plant biomass and soil fungal diversity from the linear regression (positive) and SEM (negative) analyses suggest that plant biomass is not a good predictor of soil fungal richness. Previous study showed that relationships between plant biomass and soil fungal diversity varied with the seasonal dynamics of soil fungal community composition and of plant biomass production (Hiiesalu et al. 2014). In our study, the positive correlation between soil fungal richness and plant biomass in simple linear regression might be indirectly formed by soil gradient, because soil fungal richness and plant biomass are positively associated with soil gradient.

The CWM plant height positively affects plant biomass and soil fungal diversity in sandy grassland restoration. This is agreement with that the CWM traits‐driven effects on ecosystem function and properties are very important (Petchey and Gaston 2006; Butterfield and Suding 2013; Conti and Díaz 2013). This is support for the mass ratio hypothesis (Grime 1998) and emphasizes effects of the most abundant species' traits on ecosystem function and properties (Díaz et al. 2007; Conti and Díaz 2013). Previous study has suggested that plant height is strongly linked to overall plant size as well as to competitive interactions for light (Aan et al. 2006). The shade of higher plants improves microclimatic factors such as soil temperature and humidity and enables trapping of sediments and water, thus affecting soil fungal richness in sandy grassland ecosystems.

Multitrait dispersion as expressed by FDis indirectly affected soil fungal richness through its effect on plant biomass in sandy grassland restoration. FDis was positively associated with plant biomass, which is obvious evidence to support the niche‐complementarity model (Tilman 1997). This complementarity effects imply that plant biomass strongly depends on the functional characteristics of the constituent species in sandy grasslands. Following the increase of species richness in vegetation succession occurring from the sand pioneer to low shrub communities then toward the herb‐dominated communities in sandy grassland restoration (Zuo et al. 2009), niche partitioning or facilitation among plant species allows for a more complete use of resources and therefore promotes plant biomass. So, in our study, FDis is a good predictor of plant biomass in sandy grassland ecosystems (Mouillot et al. 2011). Our study supports that plant trait approaches are the useful tool to analyze plant–soil interactions and their consequences for ecosystem function and properties (Petchey and Gaston 2006; Butterfield and Suding 2013; Lavorel 2013).

Although soil properties had a directly negative effect on fungal richness in sandy grassland restoration, the total effect of soil properties on fungal richness was positive through its indirect effect. In agreement with a link between plant richness and soil gradient, we found that soil fungal richness increased with soil gradient, supporting that changes of soil properties may also better predict soil fungal diversity (Burke et al. 2009; Prevost‐Boure et al. 2011; Siciliano et al. 2014; LeBlanc et al. 2015). Structural equation models suggest that CWM plant height and plant richness have a direct effect on soil fungal richness, which is also directly controlled by altering soil properties. This study highlights the crucial role of local variation in soil properties for regulating plant trait selection and species compositions. Our study also supports that soil gradient induced the coordinated responses of plant height and leaf economics traits (Jager et al. 2015). The community‐level leaf C:N and LDMC increase with soil gradient (Table 1), suggesting that the established dominant plants along soil gradient in sandy grassland restoration have the higher N use efficiency and conservative resource use strategy (Aerts and Chapin 2000; Kazakou et al. 2006; Milcu et al. 2014). The encroachment of dominant plants with more conservative resource use and greater nutrient retention can favor soil fungal dominance through the N‐poor litter recycling (Zeller et al. 2001; Bardgett et al. 2005).

However, our conclusions need to be taken with caution when examining how plant functional diversity affects soil fungal diversity, due to the measured accuracy of soil fungal diversity. Although DGGE method can give a good indication of general fungal population shifts across habitat gradient, its low resolution maybe underestimate soil fungal diversity than next generation sequencing (NGS) technique (e.g., Illumina MiSeq sequencing). So, further study is necessary to test the direct or indirect effects of biotic and abiotic factors on soil fungal diversity using the NGS technique.

## Conclusions

This study is one of the first to stress that plant richness, biomass, functional traits, and diversity as well as their response to soil properties need to be taken into account when soil fungal diversity is investigated in sandy grassland restoration. Our study clearly illustrates the direct and positive effects of plant richness and CWM plant height controlled by soil gradient on soil fungal richness in sandy grassland restoration. CWM plant height and functional dispersion positively and directly affect on plant biomass. These results suggest that traits of the most abundant species within communities strongly affect plant biomass and soil fungal richness, supporting the mass ratio hypothesis. Plant functional diversity may enhance the mechanism explanation of associations of soil fungal diversity with vegetation structure and soil properties in sandy grassland restoration. Thus, plant functional traits and diversity need be used to better understand how soil fungal diversity will be affected by changes of vegetation and soil in sandy grassland ecosystems. More effort should be paid to protect the sandy grasslands with the high species richness and high‐standing dominant plants in order to maintain the high soil fungal richness. Understanding the key direct and indirect drivers of soil fungal diversity and their relationships is critical for the management and restoration of sandy ecosystems subjected to similar climate and land management.

## Conflict of Interest

None declared.

## Supporting information


**Table S1.** Vegetation characteristics and soil properties at four habitats of sandy grassland (Mean ± SE).
**Table S2.** Summary of functional diversity components at four habitats of sandy grassland (Mean ± SE).
**Table S3.** Identity of the sequences on the selected DGGE bands by BLAST in NCBI.
**Table S4.** Intra‐set correlations of the soil properties, eigenvalue and cumulative percentage variance for the first two axes of principal component analysis (PCA).
**Figure S1.** Heat map of the fungal community composition based on the DGGE bands and band intensities in 24 plots along a gradient of grassland restoration.
**Figure S2.** Phylogenetic tree (Neighbor Joining) for the 18S rDNA gene sequences.
