# Supplementary material for: Plant functional diversity enhances associations of soil fungal diversity with vegetation and soil in the restoration of semiarid sandy grassland
Source: Ecol Evol. 2015 Dec 29;6(1):318–28. doi: 10.1002/ece3.1875 (PMC4716495; doi:10.1002/ece3.1875)
Supplement: Supplementary file 1 — Table S1. Vegetation characteristics and soil properties at four habitats of sandy grassland (Mean ± SE). Table S2. Summary of functional diversity components at four habitats of sandy grassland (Mean ± SE). Table S3. Identity of the sequences on the selected DGGE bands by BLAST in NCBI. Table S4. Intra‐set correlations of the soil properties, eigenvalue and cumulative percentage variance for the first two axes of principal component analysis (PCA). Figure S1. Heat map of the fungal community composition based on the DGGE bands and band intensities in 24 plots along a gradient of grassland restoration. Figure S2. Phylogenetic tree (Neighbor Joining) for the 18S rDNA gene sequences. [file ECE3-6-318-s001.docx]

Table S1. Vegetation characteristics and soil properties at four habitats of sandy grassland (Mean ± SE).

|  | MD | SFD | FD | G | F | *P* |
| --- | --- | --- | --- | --- | --- | --- |
| **Vegetation characteristics** |  |  |  |  |  |  |
| Cover (%) | 3.78±0.39 ^a^ | 44.37±1.50^b^ | 65.53±2.96 ^c^ | 72.00±2.15^d^ | 240.45 | < 0.001 |
| Species richness | 3.33±0.49^a^ | 10.83±0.75^b^ | 17.50±1.12^c^ | 13.67±1.23^d^ | 40.27 | < 0.001 |
| Biomass( g m^-2^) | 2.20±0.39^a^ | 98.10±7.27^b^ | 131.41±8.20^c^ | 190.38±19.15^d^ | 50.93 | < 0.001 |
| Belowground biomass (0-10 cm, g m^-2^) | 11.00±2.74^a^ | 112.98±25.74^b^ | 62.66±18.91^b^ | 122.52±28.97^b^ | 5.56 | < 0.01 |
| Litter mass(g m^-2^) | 2.40±1.05^a^ | 63.75±9.66^b^ | 71.88±15.93^b^ | 166.34±12.07^c^ | 37.12 | < 0.001 |
| Litter C: N | 42.78±4.52^a^ | 59.33±1.69^b^ | 41.32±2.06^a^ | 38.90±2.15^a^ | 10.76 | < 0.001 |
| **Soil properties** |  |  |  |  |  |  |
| Soil C (g kg^-1^) | 0.43±0.01^a^ | 1.10±0.08^b^ | 3.94±0.19^c^ | 5.17±0.29^d^ | 160.50 | < 0.001 |
| Soil N (g kg^-1^) | 0.11±0.01^a^ | 0.15±0.01^a^ | 0.47±0.02^b^ | 0.61±0.03^c^ | 122.00 | < 0.001 |
| C:N | 4.23±0.43^a^ | 7.56±0.30^b^ | 8.48±0.21^c^ | 8.48±0.12^d^ | 49.16 | < 0.001 |
| pH | 7.39±0.02^a^ | 7.50±0.04^a^ | 8.01±0.10^b^ | 8.47±0.06^c^ | 58.02 | < 0.001 |
| Bulk density (g cm^-3^) | 1.62±0.02^a^ | 1.57±0.01^a^ | 1.50±0.02^b^ | 1.33±0.04^c^ | 26.38 | < 0.001 |
| Electricity conductivity (μs cm^-1^) | 7.83±0.49^a^ | 11.23±0.98^a^ | 20.93±1.71^b^ | 30.00±3.41^c^ | 25.47 | < 0.001 |
| Coarse sand (2-0.25 mm, %) | 60.68±2.03^a^ | 47.01±1.55^b^ | 43.65±1.65^b^ | 21.33±4.72^c^ | 33.82 | < 0.001 |
| Fine sand (0.25-0.1 mm, %) | 37.49±2.12^ac^ | 49.73±1.31^b^ | 42.22±3.16^a^ | 35.22±2.35^c^ | 7.57 | < 0.01 |
| Very fine sand (0.1-0.05 mm, %) | 1.48±0.09^a^ | 1.85±0.27^a^ | 5.62±1.32^a^ | 31.36±5.43^b^ | 26.15 | < 0.001 |
| Silt + Clay (<0.05 mm, %) | 0.35±0.03^a^ | 1.41±0.23^a^ | 8.51±0.61^b^ | 12.09±1.77^c^ | 35.90 | < 0.001 |
| Soil water content (%) | 2.86±0.17^a^ | 2.60±0.28^a^ | 3.36±0.51^a^ | 5.01±0.53^b^ | 7.23 | < 0.01 |

MD, Mobile dune; SFD, Semi-fixed dune; FD, Fixed dune; G, Grassland; specific leaf area (SLA), leaf dry matter content (LDMC); Different letters in from mean values indicate statistical difference among different habitats at *P*<0.05.

Table S2. Summary of functional diversity components at four habitats of sandy grassland (Mean ± SE).

|  | MD | SFD | FD | G | F | *P* |
| --- | --- | --- | --- | --- | --- | --- |
| ***Community weighted means (CWM)*** | | |  |  |  |  |
| Plant height (cm) | 9.23±0.99^a^ | 24.95±2.63^b^ | 43.12±2.11^c^ | 64.80±5.81^d^ | 49.66 | < 0.001 |
| SLA (m^-2^ kg^-1^) | 16.83±1.58^a^ | 12.93±0.85^b^ | 19.12±0.56^a^ | 19.48±0.77^a^ | 8.82 | < 0. 01 |
| LDMC (g kg^-1^) | 261.12±9.76^a^ | 207.68±9.43^b^ | 270.42±11.33^a^ | 279.98±10.22^a^ | 10.03 | < 0.001 |
| Leaf C:N | 14.72±0.65^a^ | 20.11±0.96^b^ | 19.79±0.44 ^b^ | 20.23±0.87^b^ | 12.38 | < 0.001 |
| ***Multi-traits functional diversity index*** | | |  |  |  |  |
| Function dispersion | 0.02±0.01^a^ | 0.08±0.02^bc^ | 0.05±0.01^ac^ | 0.10±0.01^b^ | 8.56 | < 0.01 |

MD, Mobile dune; SFD, Semi-fixed dune; FD, Fixed dune; G, Grassland; specific leaf area (SLA), leaf dry matter content (LDMC). Different letters in from mean values indicate statistical difference among different habitats at *P*<0.05.

Table S3. Identity of the sequences on the selected DGGE bands by BLAST in NCBI

| DGGE band | Closest strain from GenBank by blast | Accession number | Similarity (%) | Phylogenetic affiliations |
| --- | --- | --- | --- | --- |
| 1 | *Parastagonospora nodorum* | KJ830777 | 99 | Ascomycota |
| 2 | *Cochliobolus sp.* | GU190186 | 100 | Ascomycota |
| 3 | *Arthrobotrys oligospora* | JQ809337 | 99 | Ascomycota |
| 4 | *Trematosphaeria heterospora* | AY016354 | 99 | Ascomycota |
| 5 | *Wallemia ichthyophaga EXF-994* | XR_657242 | 99 | Basidiomycota |
| 6 | *Hymenoscyphus kiko* | KC164672 | 99 | Ascomycota |
| 7 | *Cladosporium sp. Pt-2* | JQ824844 | 99 | Ascomycota |
| 8 | *Geomyces sp. P7* | GQ214385 | 99 | Ascomycota |
| 9 | *Cochliobolus sp. 007(L)1-1* | FJ235087 | 99 | Ascomycota |
| 10 | *Myrothecium verrucaria* | EF211127 | 100 | Ascomycota |
| 11 | *Ciboria batschiana* | KJ830799 | 98 | Ascomycota |
| 12 | *Mortierella alpina* | AY550125 | 99 | Zygomycota |
| 13 | *Graphium fructicola* | AB007659 | 91 | Ascomycota |
| 14 | *Amyloporia xantha* | KJ830780 | 100 | Basidiomycota |
| 15 | *Uncultured fungus clone L7* | DQ412118 | 99 | *Fungi incertae sedis* |
| 16 | *Pleospora herbarum* | PHU43458 | 99 | Ascomycota |
| 17 | *Salpingoeca fusiformis* | KJ631039 | 94 | *Fungi incertae sedis* |
| 18 | *Geranomyces variabilis* | AF164241S1 | 98 | Chytridiomycota |
| 19 | *Wickerhamomyces anomalus* | AY251638 | 99 | Ascomycota |
| 20 | *Dioszegia hungarica* | AF314235 | 87 | Basidiomycota |
| 21 | *Melanoleuca verrucipes* | DQ457645 | 99 | Basidiomycota |
| 22 | *Deconica montana* | DQ465342 | 99 | Basidiomycota |
| 23 | *Mortierella alpina* | AY546097 | 99 | Zygomycota |
| 24 | *Pseudogymnoascus destructans* | GU999983 | 99 | Ascomycota |
| 25 | *Saccharomyces sp. WW-W8* | DQ345290 | 98 | Ascomycota |
| 26 | *Campanella sp. MCA2235* | AY916675 | 99 | Basidiomycota |
| 27 | *Glyphium elatum* | AF346419 | 99 | Ascomycota |
| 28 | *Pseudogymnoascus destructans* | GU350433 | 100 | Ascomycota |
| 29 | *Purpureocillium lilacinum* | HQ232214 | 99 | Ascomycota |
| 30 | *Phoma sp. ZH2.1* | FJ450059 | 98 | Ascomycota |
| 31 | *Phoma macrostoma var.* | AB454217 | 99 | Ascomycota |
| 32 | *Dothideomycetes sp. LS-2013a* | KC315859 | 98 | Ascomycota |
| 33 | *Lulwoana uniseptata* | AY879034 | 96 | Ascomycota |
| 34 | *Macrolepiota dolichaula* | AY771602 | 98 | Basidiomycota |
| 35 | *Nanoscypha tetraspora* | AF006314 | 97 | Ascomycota |
| 36 | *Candida glucosophila* | AB013519 | 99 | Ascomycota |
| 37 | *Helicascus elaterascus* | AF053727 | 98 | Ascomycota |
| 38 | *Trichoderma reesei* | JN831373 | 99 | Ascomycota |

Table S4. Intra-set correlations of the soil properties, eigenvalue and cumulative percentage variance for the first two axes of principal component analysis (PCA).

|  | PCA1 | PCA2 |
| --- | --- | --- |
| Soil C (g kg^-1^) | 0.98^***^ | 0.16 |
| Soil N (g kg^-1^) | 0.97^***^ | 0.12 |
| Soil C/N | 0.74^***^ | 0.46^*^ |
| pH | 0.94^***^ | 0.08 |
| Bulk density (g cm^-3^) | -.90^***^ | 0.11 |
| Electricity conductivity (μs cm^-1^) | 0.93^**^ | 0.04 |
| Coarse sand (2-0.25 mm, %) | -0.90^**^ | 0.10 |
| Fine sand (0.25-0.1 mm, %) | -0.43 | 0.56^**^ |
| Very fine sand (0.1-0.05 mm, %) | 0.87_***_ | -0.44^*^ |
| Silt + Clay (<0.05 mm, %) | 0.96_***_ | 0.08 |
| Soil water content (%) | 0.75^***^ | -0.27 |
| Eigenvalues | 0.87 | 0.08 |
| Cumulative percentage variance (%) | **86.80** | 95.00 |

*, *P* < 0.05; **, *P* < 0.01; ***, *P* < 0.001.


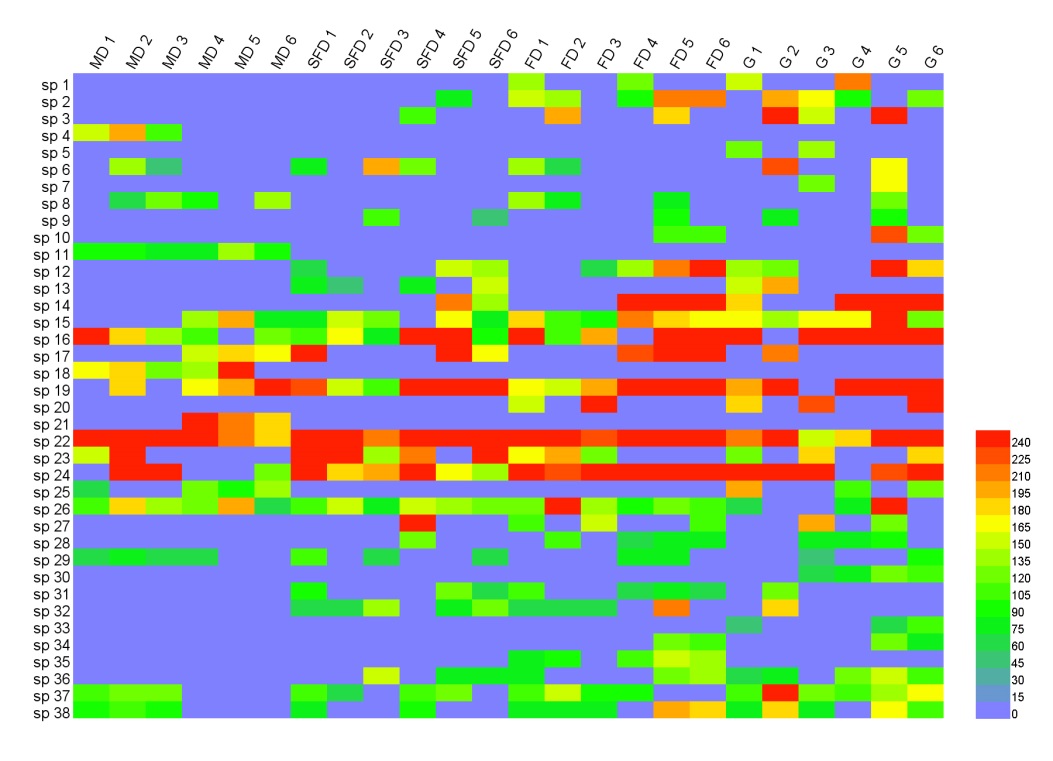


**Fig. S1** Heat map of the fungal community composition based on the DGGE bands and band intensities in 24 plots along a gradient of grassland restoration. MD: mobile dunes; SFD: semi-fixed dunes; FD: fixed dunes; G: grasslands; sp: fungal species number.


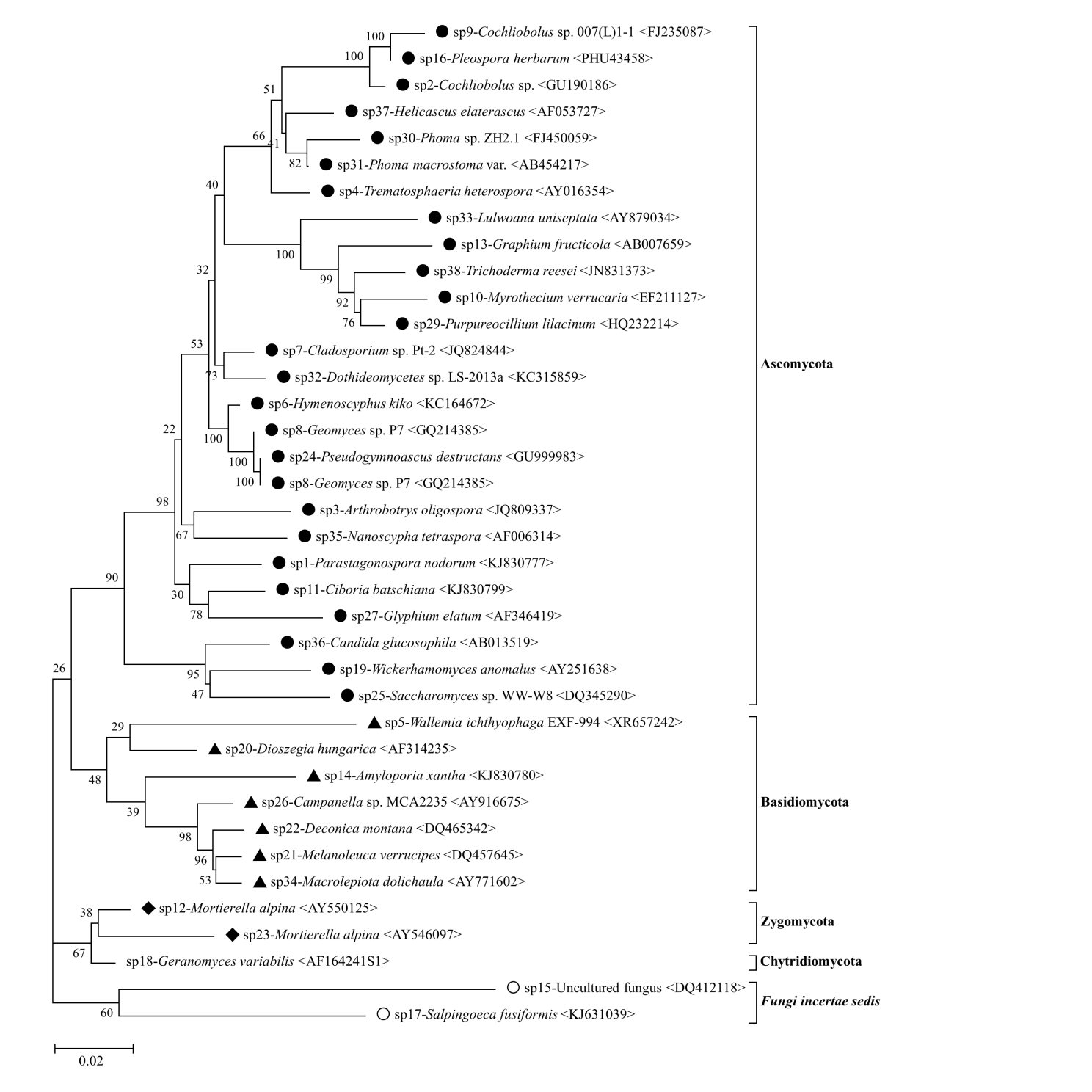
**Fig. S2.** Phylogenetic tree (Neighbor Joining) for the 18S rDNA gene sequences. Fungal species numbers (sp1-sp38) correspond to the band numbers.
